# Cedar Pollinosis and Mortality: A Population-Based Prospective Cohort Study in Japan

**DOI:** 10.2188/jea.JE20170278

**Published:** 2019-02-05

**Authors:** Kenichi Mori, Keiko Wada, Kie Konishi, Yuko Goto, Fumi Mizuta, Sachi Koda, Takahiro Uji, Yatsuji Ito, Chisato Nagata

**Affiliations:** 1 Department of Otolaryngology, Gifu University Graduate School of Medicine, Gifu, Japan; 2 Department of Epidemiology and Preventive Medicine, Gifu University Graduate School of Medicine, Gifu, Japan

**Keywords:** cedar pollinosis, allergic rhinitis, respiratory disease, mortality, cohort study

## Abstract

**Background:**

Cedar pollinosis is one of the most prevalent forms of seasonal allergic reaction in Japan. Only one prospective study has examined the association between cedar pollinosis and mortality. Using a symptom-based questionnaire on cedar pollinosis, we investigated the association of cedar pollinosis with all-cause and cause-specific mortality.

**Methods:**

Data came from the Takayama Study, which recruited residents aged ≥35 years in 1992 from Takayama city in Gifu Prefecture, Japan. The current study used information on cedar pollinosis that was obtained from the second survey in 2002. A total of 12,471 persons who were 45–80 years old and had no history of cancer, coronary heart disease, or stroke responded to a questionnaire asking about four symptoms related to cedar pollinosis. Mortality and migration data were obtained throughout the follow-up period up to March 2013. Cox proportional hazard models were used to examine the relation between cedar pollinosis and mortality.

**Results:**

A total of 1,276 persons died during follow-up period. Among these, there were 504 neoplasm, 278 cardiovascular, and 181 respiratory deaths. After adjusting for potential confounders, cedar pollinosis was associated with significantly lower all-cause mortality (hazard ratio [HR] 0.79; 95% confidence interval [CI], 0.65–0.95) and respiratory mortality (HR 0.38; 95% CI, 0.18–0.82). There was no significant association between cedar pollinosis and mortality due to neoplasm or cardiovascular disease.

**Conclusions:**

We found an inverse association between cedar pollinosis and the risk of all-cause and respiratory mortality. Further research is needed to elucidate the association between cedar pollinosis and mortality.

## INTRODUCTION

In Japan, cedar pollinosis is one of the most prevalent forms of seasonal allergic reaction caused by the pollen of the cedar tree (*Cryptomeria japonica*). In spring, many patients with cedar pollinosis experience more severe symptoms, including rhinitis and/or conjunctivitis, accompanied by pollen-specific immunoglobulin E (IgE) production for longer periods of time, compared with other pollen allergies.^1^ Cedar pollinosis patients started to appear in the 1950s, and the number of patients has increased gradually since the 1970s.^2^^,^^3^ In the nationwide epidemiological surveys conducted by the same authors, the prevalence rates of self-reported diagnosis of cedar pollinosis were 17.4% in 1998 and 26.5% in 2008.^4^^,^^5^

To our knowledge, only one prospective study has been conducted to examine whether cedar pollinosis is associated with mortality.^6^ The study indicated that people who reported having a diagnosis of cedar pollinosis were at a significantly lower risk of all-cause mortality among middle-aged and elderly Japanese. Physician-diagnosed pollinosis may largely depend on more frequent visits to the doctor and higher health consciousness. Furthermore, some people may not realize that they have this disease, despite having symptoms. Therefore, in the present study, we used a questionnaire asking about various symptoms to uniformly define cedar pollinosis and investigated the association between cedar pollinosis and all-cause and cause-specific mortality in a large cohort of Japanese men and women (the Takayama Study).

## METHODS

### Study population

Participants in this study are cohort members from the Takayama Study, which is a population-based cohort study initiated in 1992. The design and methodology of the study have been described elsewhere.^7^^,^^8^ All non-hospitalized residents aged 35 years or older in Takayama city in Gifu Prefecture, Japan were invited to participate in the study. A total of 31,552 persons (participation rate: 85.3%) completed a questionnaire on demographic characteristics, smoking and drinking habits, diet, exercise, education, and medical and reproductive histories. In July 2002, a second survey was conducted to update lifestyle and health-status information, including questions regarding cedar pollinosis. In this survey, the target population was restricted to those who were younger than 70 years old in 1992. After the exclusion of those who were deceased, physically unable to complete the questionnaire, or had relocated, the study population consisted of 22,435 individuals, of whom 14,975 (66.7%) responded to the second questionnaire.

For the present analysis, we excluded subjects who had reported a history of cancer (*n* = 629), coronary heart disease (*n* = 1,000), or stroke (*n* = 257) before the 2002 second survey, since the presence of these diseases could have affected their subsequent mortality. Furthermore, those who did not respond completely to the questions regarding cedar pollinosis (*n* = 723) were excluded. Hence, the population for the analysis consisted of 12,471 subjects (5,532 men and 6,939 women).

### Cedar pollinosis assessment

The questionnaire regarding cedar pollinosis was developed based on that reported by Endo et al.^9^ Information was sought regarding four symptoms of cedar pollinosis: sneezing, nasal discharge, nasal obstruction, and eye irritation. Cedar pollinosis was defined as the existence of three of the four symptoms, with at least one symptom occurring in spring only. Before the second survey, this questionnaire had been validated through diagnosis by an otolaryngologist based on nasal discharge, nasal provocation test, intracutaneous test, and pollen-specific IgE positivity as the gold standard. The sensitivity and specificity were 0.80 and 0.65, respectively. The details of the questionnaire and its validity have been described elsewhere.^10^

### Outcome ascertainment

The deaths and their causes were confirmed using data provided by the Legal Affair Bureau, Japan. The causes of death were coded according to the International Classification of Diseases (ICD), 10th Revision. The major endpoint of this study was all-cause mortality. We also considered disease-specific endpoints, including neoplasm (C00–D48), cardiovascular disease (I00–I99), and respiratory disease (J00–J99). Information concerning individuals who moved away from the survey area was obtained from residential registers or family registers. During the study period, 216 individuals moved out of the survey area, and the date of emigration was unknown for one subject. This study was approved by the ethics board of the Gifu University Graduate School of Medicine.

### Statistical analysis

A follow-up period was calculated for each participant from the second survey (July 1, 2002) to the date of death, the date of emigration out of Takayama, or the end of the study (March 31, 2013), whichever came first. One subject who moved away on an unknown date was censored at the latest point when he was confirmed to live in the study area. The hazard ratios (HRs) and 95% confidence intervals (CIs) of all-cause and cause-specific mortalities according to cedar pollinosis status were calculated using the Cox proportional hazard model. The multivariate analysis was conducted using the covariates of age (years, continuous), sex (male or female), body mass index (BMI; <18.5, 18.5–24.9, 25.0–29.9, or ≥30.0 kg/m^2^), physical activity score (continuous), smoking status (never, former, current little; ≤20 cigarettes/day, current much; or >20 cigarettes/day), alcohol consumption (<23, 23–46, or >46 g/day), education level (<8, 9–11, 12–14, or ≥15 years), marital status (married or not married), and medical history of diabetes and hypertension (yes or no). Physical activity was assessed by asking participants the average number of hours they had spent weekly to carry out various kinds of activities during the last year in a validated questionnaire, translated into a metabolic equivalent (MET), and summed to obtain a physical activity score (METs-h/week).^11^ Individuals missing information for these covariates were assigned an indicator variable in the analysis. As for alcohol consumption and education level, information from the first survey was used because we did not obtain those data in the second survey. The information on diet, including alcohol consumption, was derived from a food frequency questionnaire that had already been validated.^12^

For sensitivity analyses, we repeated the analyses after cedar pollinosis was redefined as a condition in which all four symptoms appeared in the spring. The sensitivity and specificity for the revised definition of cedar pollinosis were 0.32 and 0.98, respectively. All of the statistical analyses were performed using SAS programs (SAS Institute, Cary, NC, USA). Significance was defined as two-sided *P* < 0.05.

## RESULTS

At the second survey, 19.6% of respondents (2,444 of 12,471) had cedar pollinosis. Table 1 shows the characteristics of the participants according to cedar pollinosis status. The mean ages of participants with and without cedar pollinosis were 57.5 (standard deviation [SD], 8.0) and 62.8 (SD, 8.9), respectively. On average, participants with cedar pollinosis were more likely to be younger, female, never smokers, and more educated than those without cedar pollinosis.

**Table 1.** Characteristics of the Takayama cohort participants at the time of the 2002 second survey

Characteristic	Participants with cedar pollinosis		Participants without cedar pollinosis	
	(*n* = 2,444)		(*n* = 10,027)	
	
	*n*	%	*n*	%
Sex				
Male	960	39.3	4,572	45.6
Female	1,484	60.7	5,455	54.4
Age, years				
45–54	1,148	47.0	2,447	24.4
55–64	831	34.0	3,304	33.0
65–74	403	16.5	3,276	32.7
75–80	62	2.5	1,000	10.0
Body mass index, kg/m^2^				
14–18.4	139	5.7	733	7.3
18.5–24.9	1,866	76.4	7,524	75.0
25–29.9	384	15.7	1,497	14.9
30–40	31	1.3	90	0.9
Smoking status				
Never	1,549	63.4	5,640	56.2
Former	518	21.2	1,843	18.4
Current little	291	11.9	1,877	18.7
Current much	76	3.1	606	6.0
Alcohol consumption, g/day				
<23	1,723	70.5	6,671	66.5
23–46	321	13.1	1,389	13.9
>46	400	16.4	1,967	19.6
Education, years				
<8	136	5.6	1,620	16.2
9–11	897	36.7	4,133	41.2
12–14	1,143	46.8	3,409	34.0
>15	248	10.1	792	7.9
Married	2,185	89.4	8,384	83.6
History of hypertension	492	20.1	2,459	24.5
History of diabetes	127	5.2	665	6.6

During the 10.75-year follow-up period, a total of 1,276 participants died from all causes. Among these, there were 504 neoplasm, 278 cardiovascular, and 181 respiratory deaths (Table 2). Cedar pollinosis was associated with significantly lower all-cause mortality after controlling for age and sex; the HR was 0.75 (95% CI, 0.62–0.91) among those with cedar pollinosis compared with those without cedar pollinosis. After adjusting for all covariates, all-cause mortality remained statistically significant (HR 0.79; 95% CI, 0.65–0.95). Stratified analyses according to sex showed that the HRs of all-cause mortality among participants with cedar pollinosis were 0.81 (95% CI, 0.64–1.03) in men and 0.75 (95% CI, 0.55–1.04) in women. There was no significant association between cedar pollinosis and mortality due to neoplasm or cardiovascular disease. Cedar pollinosis was associated with a significantly reduced HR (0.38; 95% CI, 0.18–0.82) for death from respiratory disease.

**Table 2.** Hazard ratios of all-cause and cause-specific mortality according to the cedar pollinosis status in the Takayama study, July 2002–March 2013

Cause of death		Number of deaths	Age & sex adjusted		Multiple adjusted^*^	
	
			HR	95% CI	HR	95% CI
All causes	Without CP	1,155	1.00		1.00	
	With CP	121	0.75	0.62–0.91	0.79	0.65–0.95
Neoplasms	Without CP	446	1.00		1.00	
	With CP	58	0.82	0.62–1.09	0.88	0.66–1.16
Cardiovascular diseases	Without CP	250	1.00		1.00	
	With CP	28	0.92	0.62–1.37	0.95	0.64–1.41
Non-neoplastic, non-cardiovascular	Without CP	459	1.00		1.00	
	With CP	35	0.58	0.41–0.81	0.60	0.42–0.85
Respiratory diseases	Without CP	174	1.00		1.00	
	With CP	7	0.37	0.17–0.79	0.38	0.18–0.82

CI, confidence interval; CP, cedar pollinosis; HR, hazard ratio.

^*^Adjusted for age, sex, BMI, physical activity, smoking status, alcohol consumption, education, marital status, and medical history of diabetes and hypertension.

*n* = 25,549 person-years for participants with cedar pollinosis and *n* = 101,937 person-years for participants without cedar pollinosis.

When we changed the definition of cedar pollinosis to one including all four symptoms occurring during spring, 976 respondents (7.8%) had cedar pollinosis. The decreased risk of all-cause mortality was also observed among people with cedar pollinosis (HR 0.58; 95% CI, 0.39–0.86). No one died from respiratory disease in the cedar pollinosis group.

Similar trends were also observed when we included subjects who had reported a history of cancer, coronary heart disease, or stroke. Cedar pollinosis was associated with a significantly decreased risk of all-cause mortality (HR 0.84; 95% CI, 0.72–0.99) and respiratory mortality (HR 0.56; 95% CI, 0.32–0.96). There was no significant association between cedar pollinosis and mortality due to neoplasm or cardiovascular disease.

## DISCUSSION

Using a symptom-based questionnaire on cedar pollinosis, we found that cedar pollinosis was associated with decreased risks of all-cause mortality and mortality due to respiratory disease in this population-based cohort. A previous prospective cohort study among 8,796 Japanese men and women found that subjects’ self-report of pollinosis was associated with significantly reduced risks of all-cause mortality and death from neoplasms.^6^ The multiple adjusted HRs of pollinosis for all-cause and neoplasm mortalities were 0.57 (95% CI, 0.38–0.87) and 0.48 (95% CI, 0.26–0.92), respectively. Since there were no respiratory deaths in the pollinosis group, the association between pollinosis and respiratory deaths could not be assessed in that study. In this study, we confirmed an inverse association between pollinosis and all-cause mortality using the different definition of pollinosis status. In addition, for the first time, we detected a significantly lower risk of dying from respiratory disease among people with cedar pollinosis than those without cedar pollinosis.

Cedar pollinosis is classified as seasonal allergic rhinitis.^13^ Sakurai et al estimated that 81.0% of the allergic rhinitis reported by study participants was seasonal rhinitis, with symptoms evoked from February to May (the months of cedar pollination) in Japan.^14^ Although allergic rhinitis represents a global healthcare problem,^15^ to our knowledge, only two studies have examined the association between allergic rhinitis and mortality.^16^^,^^17^ One study, the first National Health and Nutrition Examination Survey Epidemiologic Follow-Up Study, did not observe that hay fever, also known as an allergic rhinitis, was associated with mortality.^16^ Another study among the American population found that patients with physician-diagnosed allergic rhinitis had a significantly lower risk of all-cause mortality,^17^ which is similar to our results.

We have no immediate explanation for the potential mechanism underlying the observed inverse association between cedar pollinosis and all-cause and respiratory mortality. However, it has been reported that nasal mucus acts as a barrier against external pathogens and has antioxidant, antiprotease, and antimicrobial activities.^18^ Mucus from patients with allergic rhinitis showed an enhanced immune response.^19^ These findings suggest that cedar pollinosis might create a favorable immunological environment, which led to a decrease in the risk of respiratory mortality as well as all-cause mortality. The airway epithelium is thought to play a key role in the regulation of airway immune response.^20^ The environments of airway epithelium, such as phagocytes and cytokines in the secretions, also may have favorable profile to preserve the lung function in people with cedar pollinosis. Nonetheless, this is the first study to observe a significant inverse association between cedar pollinosis and respiratory mortality, and our findings require confirmation in additional study.

The strengths of our study include its prospective population-based design, a long follow-up period, and information obtained for several confounders. However, the present study has several limitations. Although we used the symptom-based questions about cedar pollinosis to reduce any selection bias that would occur using physician diagnosis, we cannot fully exclude the possibility that persons defined as having pollinosis were health-conscious, which might have contributed to the reduction in mortality. Additionally, considering the relatively low specificity of the questionnaire, some participants may be misclassified as “cases”. However, the relationship between cedar pollinosis and mortality was not essentially altered after we adopted a definition with low sensitivity and high specificity. We accounted for past alcohol consumption, but not current consumption, in the second survey. Although the individuals’ drinking habits may have changed since the first survey, the reliability of drinking at intervals of 4.5 years was reported to be excellent (Kappa value: 0.85).^21^ Thus, it is unlikely that the association between pollinosis and mortality would have been largely changed by the adjustment for past alcohol consumption. The response rate in the present study was not very high, and our results were derived from only one community. Therefore, these results might not be readily generalized to the whole of Japan. In addition, the sample size was too small to permit a more detailed division of causes of death, which might have permitted us to gain a better understanding of the observed associations.

In conclusion, we found an inverse association between cedar pollinosis and all-cause and respiratory mortality. Although our findings suggest that cedar pollinosis might have implications for longevity, further research is needed to evaluate the impact of cedar pollinosis on mortality and, thereafter, to determine the possible mechanisms underlying the association between cedar pollinosis and mortality.

## References

[1] Yamada T, Saito H, Fujieda S Present state of Japanese cedar pollinosis: the national affliction. J Allergy Clin Immunol. 2014;133:632–639.e5. 10.1016/j.jaci.2013.11.002 24361081

[2] Kishikawa R, Hirose T, Nishima S. *Report on the prevention and treatment of pollinosis*. Ministry of Health and Welfare; 1990:43–48 [in Japanese].

[3] Saito Y Japanese cedar pollinosis: discovery, nomenclature, and epidemiological trends. Proc Jpn Acad, Ser B, Phys Biol Sci. 2014;90:203–210. 10.2183/pjab.90.203 24919759 PMC4160535

[4] Nakamura A, Asai T, Yoshida K, Baba K, Nakae K National epidemiological surveys on allergic rhinitis—in otorhinolaryngologists and their family members in Japan. J Otolaryngol Jpn. 2002;105:215–224.

[5] Nakae K, Baba K Update on epidemiology of pollinosis in Japan: changes over the last 10 years. Clin Exp Allergy Rev. 2010;10:2–7. 10.1111/j.1472-9733.2010.01148.x

[6] Konishi S, Ng CF, Stickley A, Watanabe C Pollinosis and all-cause mortality among middle-aged and elderly Japanese: a population-based cohort study. Clin Exp Allergy. 2016;46:1083–1089. 10.1111/cea.12638 26366720

[7] Shimizu H. *The Basic Report on Takayama Study*. Gifu, Japan: Department of Public Health, Gifu University School of Medicine; 1996.

[8] Nagata C, Takatsuka N, Shimizu H Soy and fish oil intake and mortality in a Japanese community. Am J Epidemiol. 2002;156:824–831. 10.1093/aje/kwf118 12397000

[9] Endo T. *Project report of the cedar pollinosis study for Science and Technology Agency*. 2000:20–47.

[10] Nagata C, Nakamura K, Fujii K, Smoking and risk of cedar pollinosis in Japanese men and women. Int Arch Allergy Immunol. 2008;147:117–124. 10.1159/000135698 18520156

[11] Suzuki I, Kawakami N, Shimizu H Reliability and validity of a questionnaire for assessment of energy expenditure and physical activity in epidemiological studies. J Epidemiol. 1998;8:152–159. 10.2188/jea.8.152 9782671

[12] Shimizu H, Ohwaki A, Kurisu Y, Validity and reproducibility of a quantitative food frequency questionnaire for a cohort study in Japan. Jpn J Clin Oncol. 1999;29:38–44. 10.1093/jjco/29.1.38 10073150

[13] Okubo K, Kurono Y, Fujieda S, ; Japanese Society of Allergology Japanese guideline for allergic rhinitis. Allergol Int. 2011;60:171–189. 10.2332/allergolint.11-RAI-0334 21636965

[14] Sakurai Y, Nakamura K, Teruya K, Prevalence and risk factors of allergic rhinitis and cedar pollinosis among Japanese men. Prev Med. 1998;27:617–622. 10.1006/pmed.1998.0336 9672957

[15] Bousquet J, Schünemann HJ, Samolinski B, ; World Health Organization Collaborating Center for Asthma and Rhinitis Allergic Rhinitis and its Impact on Asthma (ARIA): achievements in 10 years and future needs. J Allergy Clin Immunol. 2012;130:1049–1062. 10.1016/j.jaci.2012.07.053 23040884

[16] Savage JH, Matsui EC, McCormack M, Litonjua AA, Wood RA, Keet CA The association between asthma and allergic disease and mortality: a 30-year follow-up study. J Allergy Clin Immunol 2014;133:1484–1487.e1-5 10.1016/j.jaci.2014.01.028 24636085 PMC4006089

[17] Crans Yoon AM, Chiu V, Rana JS, Sheikh J Association of allergic rhinitis, coronary heart disease, cerebrovascular disease, and all-cause mortality. Ann Allergy Asthma Immunol. 2016;117:359–364.e1. 10.1016/j.anai.2016.08.021 27742084

[18] Voynow JA, Rubin BK Mucins, mucus, and sputum. Chest. 2009;135:505–512. 10.1378/chest.08-0412 19201713

[19] Tomazic PV, Birner-Gruenberger R, Leitner A, Obrist B, Spoerk S, Lang-Loidolt D Nasal mucus proteomic changes reflect altered immune responses and epithelial permeability in patients with allergic rhinitis. J Allergy Clin Immunol. 2014;133:741–750. 10.1016/j.jaci.2013.09.040 24290289

[20] Yaghi A, Dolovich MB Airway epithelial cell cilia and obstructive lung disease. Cells. 2016;5(4):40. 10.3390/cells5040040 27845721 PMC5187524

[21] Zhu S, Toyoshima H, Kondo T, Short- and long-term reliability of information on previous illness and family history as compared with that on smoking and drinking habits in questionnaire surveys. J Epidemiol. 2002;12:120–125. 10.2188/jea.12.120 12033522 PMC10468344

